# Prostate cancer and glutathione S-transferase deletions

**DOI:** 10.17179/excli2015-192

**Published:** 2015-09-21

**Authors:** Saima Shakil Malik, Nosheen Masood, Azra Yasmin

**Affiliations:** 1 Fatima Jinnah Women University, The Mall, Rawalpindi, Pakistan, E9, Environmental Sciences Department/ Biotechnology

**Keywords:** GSTM1, GSTT1, polymerase chain reaction (PCR), prostate cancer

## Abstract

*GSTM1* and *GSTT1* gene polymorphisms have been studied in many populations to evaluate their association with prostate cancer risk with contrasting results. The current study was aimed to find out the association of *GSTM1* and *GSTT1* gene polymorphisms with prostate cancer in Pakistani men. This case control study included pathologically confirmed prostate cancer patients and age matched male controls. Epidemiological data was collected by a standard questionnaire and presence or absence of *GSTM1* and *GSTT1 *gene was observed by multiplex PCR using CYP1A1 as housekeeping gene. Prostate cancer was more prevalent in age of >60 years and most of the patients were at stage IV (70 %) and have undergone surgery. Family history of cancer, smoking, metastasis and surgery were found to be significant (P<0.05) risk factors in prostate cancer development. Gleason score 7 was most prevalent (40.5 %) in prostate cancer patients. Source of drinking water, residential area, occupation, eating habits and number of family members had no association (P>0.05) with prostate cancer risk. No significant association was found when comparing *GSTM1 *(OR=0.78) and *GSTT1* (OR=0.89) gene deletions with prostate cancer risk. Smoking and TNM staging were also not associated with deletion of *GSTM1* and *GSTT1* genes. Comparison of dual null deletion of both genes with prostate cancer also showed non-significant associations. Deletion of *GSTM1* gene at stage IV prostate cancer patients was significantly higher compared with other stages of cancer while no significance was shown by *GSTT1* gene deletion. *GSTM1, GSTT1 *and deletion of both *GSTM1* and *GSTT1* genes do not contribute towards increased risk of prostate cancer in Pakistani population.

## Introduction

Prostate cancer is one of the most commonly diagnosed cancer in the Western world. The incidence rate of prostate cancer reported among Asians is much lower as compared to Americans (Yang et al., 2013[24]). In Pakistan it is the third frequent malignancy in males and the diagnostic and therapeutic facilities of such a prevalent cancer are limited in our country (Ahmad et al., 2009[2]). In order to understand the biology of prostate cancer and develop new interventions, complete analysis of different risk factors along with their comparison with genetics is essential (Hoffman, 2011[10]). 

Epidemiological data suggested that age, smoking, dietary habits, genetics and many other factors may be involved in prostate cancer development. Age plays an important role in the development of prostate cancer and its incidence increases with the increased age. An abrupt increase in the prostate cancer cases was observed in the last two to three decades in Pakistan because of increased industrialization and urbanization (Mahmood et al., 2012[16]). Common symptoms for prostate cancer includes frequent urination, dysuria, hematuria, nocturia and difficulty in maintaining steady urine stream (Cramer et al., 2007[9]).

Glutathione S-transferases (GSTs) are detoxifying enzymes and play an important role in the detoxification of a number of exogenous and endogenous carcinogens by conjugation with glutathione (Agalliu et al., 2006[1]). Deletion of *GSTM1 *and *GSTT1 *genes leads to failure of the expression of GST proteins, which may cause reduced detoxification of potential carcinogens and lead to greater vulnerability to cancer (Spurdle et al., 2001[22]). 

Recently, several studies have evaluated the association of *GSTM1* and *GSTT1* with prostate cancer but inconsistency is observed in the results (Albertsen et al., 2005[3]; Hu et al., 2013[11]; Yang et al., 2013[24]). However no specific data is available for Pakistani population regarding their genetic status. Therefore, current study is aimed to evaluate the xenobiotic metabolizing gene expression (*GSTM1 *and* GSTT1*) at DNA level and find out their correlation with progression of prostate cancer along with environmental risk factors. 

## Materials and Methods

This case control study was conducted over a period of 12 months and included 350 prostate cancer patients and 300 controls. Blood sample from already diagnosed and undergoing treatment prostate cancer patients were collected from Benazir Bhutto Shaheed Hospital, Rawalpindi, Shifa International Hospital, Urology department, Islamabad, Nuclear Medicine Oncology and Radiotherapy Institute, Islamabad and Institute of Nuclear Medicine and Oncology Lahore. Whereas control samples were collected from general population from Lahore, Rawalpindi and Islamabad. 

Inclusion criteria for cases was histologically diagnosed and confirmed by oncologists as prostate cancer patients with Gleason scores 5-10 and for controls it was that the individuals should be cancer free normal at the time of sampling and they should not have any family history of cancer and PSA level was normal (PSA < 4 ng/dl). Epidemiological data was collected by a standard questionnaire. Study was approved from the ethical committees of university as well as hospitals. Informed consent was taken from controls and patients before collecting any type of information or sample. DNA was extracted from lymphocytes by using phenol chloroform method and *GSTM1 *and* GSTT1* was analyzed by multiplex PCR (Masood et al., 2011[17]). PCR product was run on 2 % agarose gel and visualized in gel documentation system. Gels were read by two technicians blind folded to each others decision. Statistical analysis was performed by using SPSS software. 

## Results

### Environmental factors

The mean age of prostate cancer patients was 67 (+11.3) years while that of controls was 64.3 (+14.17) years. The disease was more common (86 % patients) in age of >60 years. About 8 % patients had family history of cancer (11 patients father, 9 patients grandfather and 8 patients brother had prostate cancer). As far as general symptoms associated with prostate cancer were concerned, 96 % patients had lower urinary tract symptoms (LUTS) voiding, urgency and pain in lower back showing significant association with prostate cancer (*p*<0.05). Significantly increased (*p*<0.05) levels of PSA were observed among approximately 90 % prostate cancer (CaP) patients. More than 40 % patients had bone ache and hematuria while 10 % patients were victim of fever, nocturia and sleep apnea as well. Prostate cancer patients were belonging to different occupations i.e., 30 % were living a retired life, 20 % were farmers, 10 % were bankers and 8 % were taxi drivers. Remaining 32 % prostate cancer patients were working in various fields like engineering, education, army or business persons. It was found that incidence of prostate cancer was not significantly (*p*>0.05) associated with any specific occupation. Family history of cancer (p=0.03), naswar chewing (p=0.01), cigarette (p=0.001) and huqqa smoking (p=0.02) were found to be significant risk factors in prostate cancer development. Gleason score 7 was (40.5 %) more common in prostate cancer patients. Source of drinking water, residential area, occupation, eating habits and number of family members had no association (*p*>0.05) with prostate cancer risk.

### GSTM1 deletion

**Figure 1:**
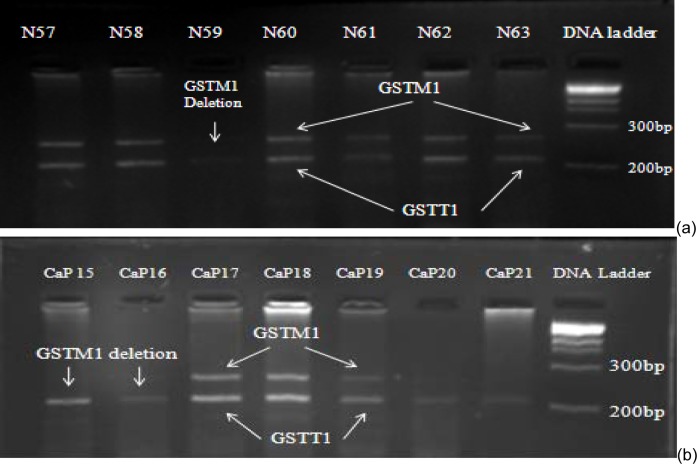
PCR products of prostate cancer patients run on 2 % agarose gel. Arrows indicate presence or deletion of *GSTM1* and *GSTT1* genes among controls (a) and prostate cancer patients (b).

*GSTM1* was deleted among 46 % control and 52 % prostate cancer patients (Figure 1(Fig. 1)). No significant association was found when comparing *GSTM1 *gene deletions (OR 0.78, CI; 0.3-1.5) with risk of prostate cancer. Statistically significant (OR 11.11; CI 3.0-40.3) number of the patients were at stage IV of prostate cancer and showed *GSTM1* gene deletion when compared with stage I and III. The results showed no significant association of *GSTM1* (*p*=0.77) deletion with smoking. 

### GSTT1 deletion

It was found that *GSTT1* gene deletion was less in patients and controls as compared with *GSTM1* (Figure 1(Fig. 1)). *GSTT1 *gene deletion was found to be non-significantly associated with prostate cancer risk (OR=0.89; CI 0.6-1.2). The results showed no significant association of *GSTT1 *deletion (*p*=0.81) with smoking. 

### Both GSTM1 and GSTT1 deletions

Null deletion of both *GSTM1* and *GSTT1* was found in 20 % and 22 % control population and prostate cancer cases respectively. The results show no significant association of both *GSTM1* and *GSTT1* deletion (OR=0.9; CI 0.6-1.3). Even no association was observed among the deletion of both genes (*p*=0.80) with smoking (Table 1(Tab. 1)).

## Discussion

It was found that occurrence of prostate cancer increases many times with increased age as reported in populations of Africa, UK, America, Australia, India and Pakistan (Bhurgri et al., 2009[5]). No significant association of prostate cancer with occupation was found (Choubey et al., 2012[8]) whereas prostate cancer and smoking were found to be strongly associated in this study as well as in African, Americans and Chinese populations (Leitzmann and Rohrmann, 2012[15]; Islami et al., 2014[12]). Such cases were also reported where number of cigarettes smoked per day were significantly associated with onset and proliferation of prostatic carcinoma. Both environment and genetics were involved in prostate cancer development. Various environmental constituents weaken the immune system and played an important role in the proliferation of cancer. A study conducted in two different islands Guadeloupe and Martinique by Belpomme in 2009 revealed that development of prostate cancer was independent of change in mens life style (Belpomme et al., 2009[4]).

Glutathione S-transferases were the most commonly studied antioxidant enzymes which were involved in the detoxification of various carcinogens and reactive oxygen species. Any change in these enzymes caused the loss of complete enzymatic activity. Hence, individuals were at greater risk towards the development of prostatic carcinoma (Mo et al., 2009[19]). 

No association was observed between *GSTM1* deletion and prostate cancer. Similar results have frequently been reported in South Indian, Japanese, American and Africans populations (Caceres et al., 2005[6]). *GSTT1* deletion was not associated with prostate cancer in the current study and in Asian, Caucasian, Korean, African and American men (Cai et al., 2014[7]). Dual null deletion of *GSTM1 *and* GSTT1* were not associated with prostate cancer and similar results had been found in studies of African descent, Brazilian and Caucasians (Lavender et al., 2009[14]). Therefore, these two genes vary ethnically and geographically. Another meta-analysis showed no association of these two genes with the development of prostate cancer (Ntais et al., 2005[20]). 

No significant association was found between the deletion of *GSTM1* and prostate cancer among smokers. Similar kind of results were revealed among Asians, North Indians and Chileans (Mittal et al., 2004[18]; Caceres et al., 2005[6]; Silig et al., 2006[21]; Wei et al., 2012[23]).

*GSTT1* deletion was also not associated with prostatic carcinoma among smokers. It was reported that *GSTT1* deletion and prostate cancer were not associated among smokers in Turkish, Finnish and Indian populations (Kidd et al., 2003[13]; Mittal et al., 2004[18]; Silig et al., 2006[21]).

## Conclusion

It was included that smoking, family history of cancer and *GSTM1* deletion at stage IV are significant risk factors of prostate cancer. However, *GSTM1, GSTT1 *and deletion of both *GSTM1* and *GSTT1* genes do not contribute towards increased risk of prostate cancer in Pakistani population. 

## Acknowledgement

All authors would like to thank the individuals and hospitals who participated in this research. Acknowledgement also goes to the University and Higher Education Commission of Pakistan for supporting this research.

## Conflict of interest

All authors declare that there is no conflict of interests in this research. No particular grant or funding was received for the project.

## Figures and Tables

**Table 1:**
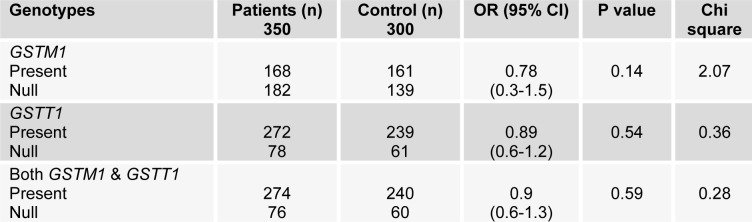
GST genotypes and prostate cancer susceptibility
